# Host Specificity of Parasitoid Wasps and Its Applications in Biological Control

**DOI:** 10.3390/insects17030244

**Published:** 2026-02-26

**Authors:** Alessandra Marieli Vacari

**Affiliations:** Laboratory of Entomology, University of Franca (UNIFRAN), Avenida Dr Armando Sales de Oliveira, 201, Parque Universitário, Franca 14404-600, SP, Brazil; alessandra.vacari@unifran.edu.br

Parasitoid wasps are among the most effective and ecologically sound natural enemies used in biological control programs. Their success is largely determined by host specificity, which enables selective suppression of target pests while minimizing risks to non-target organisms and maintaining ecological balance. Understanding the mechanisms underlying host use, host quality, parasitoid performance, and environmental compatibility is therefore fundamental for advancing sustainable pest management.

This Special Issue, “Host Specificity of Parasitoid Wasps and Its Applications in Biological Control”, brings together six studies that collectively explore host specificity from complementary ecological, physiological, and applied perspectives. Together, they demonstrate how host selection, parasitoid fitness, mass rearing strategies, multitrophic interactions, and environmental stressors shape the success of parasitoid-based biological control.

At the physiological and multitrophic levels, Aamer et al. [Contribution 1] examined the combined effects of the ectoparasitoid *Bracon hebetor* and the entomopathogenic nematode *Steinernema carpocapsae* on *Galleria mellonella*, revealing complex host stress responses, oxidative damage, and reduced survival under combined natural enemy pressure. Their findings highlight how interactions among biological control agents can influence host regulation and may enhance suppression outcomes when used compatibly.

From a production standpoint, Ramos et al. [Contribution 2] addressed a key operational challenge for augmentative biological control: reliable mass rearing. Their study demonstrated that *Telenomus podisi* reared from cryopreserved host eggs maintained comparable biological quality and performance to individuals produced from fresh eggs, supporting cryopreservation as a practical tool to improve synchronization and scalability in parasitoid production systems. Similarly, Cabral et al. [Contribution 3] evaluated field-collected and long-term laboratory populations of *Cotesia flavipes*, showing that the introduction of wild genetic material can initially enhance performance but stabilizes after several generations in captivity. These studies collectively underscore the importance of quality control and adaptive rearing strategies to maintain parasitoid efficacy in biofactories.

Host specificity and ecological safety—central pillars of classical biological control—are directly addressed by Ramadan et al. [Contribution 4], who conducted rigorous host-range testing of *Aprostocetus nitens*, a candidate parasitoid for the invasive erythrina gall wasp in Hawaiʻi. Their results demonstrate a high degree of specificity and suggest that the parasitoid may complement established agents, illustrating how careful host-range assessment can ensure both effectiveness and environmental safety.

At the community level, Li et al. [Contribution 5] documented the parasitoid complex associated with the jujube gall midge *Dasineura jujubifolia* in arid orchards of Xinjiang, identifying multiple species and revealing strong phenological synchrony between the dominant parasitoid and host population peaks. Such ecological insights are critical for conservation biological control and for optimizing release timing within integrated pest management (IPM) programs.

Finally, Wang et al. [Contribution 6] broaden the discussion to global change biology by evaluating how simulated heatwaves affect two congeneric parasitoids. Their findings demonstrate that even short-term daily heat events can reduce brood size and alter developmental performance, emphasizing that climate variability may influence parasitoid effectiveness and, consequently, trophic regulation. These results highlight the need to incorporate environmental stress tolerance into biological control planning.

Taken together, the contributions in this Special Issue reinforce that host specificity is not merely a taxonomic attribute but a dynamic ecological property shaped by host quality, rearing practices, multitrophic interactions, and environmental conditions. Advances in these areas strengthen the reliability, safety, and sustainability of parasitoid-based biological control and support their integration into modern IPM programs aimed at reducing pesticide dependence and enhancing ecosystem resilience.

We hope this Special Issue stimulates further interdisciplinary research connecting behavioral ecology, physiology, mass production technologies, and applied pest management. Such integrative approaches will be essential to fully realize the potential of parasitoid wasps as cornerstone agents in sustainable agriculture.

## Data Availability

No datasets were generated or analyzed.

